# Mitochondrial protein MPV17 promotes β-cell apoptosis in diabetogenesis

**DOI:** 10.1042/CS20230164

**Published:** 2023-08-10

**Authors:** Qiaoli Tang, Wanting Shi, Ming Liu, Liqin Tang, Wei Ren, Shaolin Shi

**Affiliations:** 1 Department of Nephrology, the First Affiliated Hospital of USTC, Division of Life Sciences and Medcine, University of Science and Technology of China, Hefei, China; 2 National Clinical Research Center for Kidney Diseases, Nanjing University School of Medicine, Nanjing, China; 3 Department of Endocrinology and Metabolism, Tianjin Medical University General Hospital, Tianjin, China; 4 Department of Medicine, Icahn School of Medicine at Mount Sinai, New York, U.S.A.

**Keywords:** apoptosis, beta-cell, diabetes, MPV17

## Abstract

MPV17 is a mitochondrial inner membrane protein, and its deficiency can cause mitochondrial DNA (mtDNA) depletion, increase reactive oxygen species (ROS), and promote apoptosis in several cell types, suggesting that MPV17 plays a protective role in cells although the underlying mechanism remains unknown. To test whether MPV17 is also protective in diabetic kidney disease, we treated* Mpv17*-deficient mice with streptozotocin (STZ) and surprisingly found that they were resistant to diabetes. *Mpv17* deficiency was also found to confer resistance to the diabetes induced by an insulin mutation (Ins2^Akita^), which represents a mouse model of monogenic diabetes characterized by proinsulin misfolding and β-cell failure. In both STZ and Ins2^Akita^ models, *Mpv17* mutants had significantly less severe β-cell loss and apoptosis compared with the wild-type mice. We next showed that MPV17 is expressed in β-cells of mice normally, suggesting that MPV17 acts β-cells autonomously to facilitate apoptosis. Consistently, Mpv17 knockdown improved the viability and ameliorated the apoptosis of cultured MIN6 cells treated with STZ and palmitic acid (PA), respectively, accompanied by prevention of caspase 3 activation. The proapoptotic effect of MPV17 in β-cells is in contrast with its known anti-apoptotic effect in other cell types. Thus, we have identified a novel regulator of β-cell death in diabetes development.

## Introduction

Diabetes is a common and prevalent disease worldwide. Type 1 diabetes is caused by insulin deficiency due to β-cell loss in the pancreatic islets. In human, Type 1 diabetes is typically an autoimmune disease in which autoreactive T cells infiltrate in the islets and destruct β-cells [1], and the autoimmunity may be initiated by β-cell abnormalities [2]. Type 2 diabetes derives from insulin-resistance of cells that metabolize glucose and can be facilitated by β-cell dedifferentiation and dysfunction [3–5]. Apoptosis is the main cause of β-cell death in Type 1 diabetes and some forms of monogenic diabetes, e.g., Wolfram syndrome, PNDM, and MODY [6–8], while the molecular mechanism underlying β-cell apoptosis is complicated and has not been fully elucidated [9].

Diabetes can affect the heart, eyes, and kidneys of the patients, and may cause other complications [10]. To study diabetic complications, several diabetic mouse models are often used. In the multiple low-dose streptozotocin (STZ) model, STZ is selectively taken up by β-cells through the highly expressed Glut2 glucose transporter on the cells. STZ alkylates genomic DNA of β-cells and induces β-cell injury, which results in islet infiltration of immune cells, including T cells and macrophages [11]. The infiltrated immune cells may worsen β-cell damage, but this effect could be limited as the immune defective SCID mice that lack immune cell infiltration in pancreatic islets in STZ treatment also develop diabetes [12]. In addition to the multiple low-dose STZ treatment, high-dose STZ is also used to induce diabetes on mice [13]. STZ-induced diabetes is characterized by β-cell apoptosis [14]. Ins2^Akita^ is a diabetes mouse model of mutant INS-gene induced diabetes of youth (MIDY) [15], in which a spontaneous Cys96Tyr mutation in insulin 2 results in protein misfolding [16]. The misfolded insulin 2 accumulates in endoplasmic reticulum (ER), resulting in ER stress and β-cell death [17]. Since ER stress is involved in β-cell pathogenesis [18], the Ins2^Akita^ may represent an ideal animal model for mechanistic study of β-cell injury in diabetogenesis.

*Mpv17* was first identified by a genetic screening of functionally essential genes in mice using retroviral insertional mutagenesis [19]. The mice with disrupted *Mpv17* gene developed proteinuria and glomerular injury. MPV17 protein is localized in mitochondrial inner membrane [20]. Several mutations in Mpv17 have been identified in an inherited disease, the mitochondrial (mt) DNA depletion syndromes (MDDS) [21], suggesting that MPV17 is required for mitochondrial DNA maintenance in certain cell types. In addition, MPV17 has been implicated in ROS generation as MPV17 deficiency can cause elevation of ROS level [22,23]. However, the mechanisms underlying the functional effects of MPV17 are largely unknown.

Interestingly, the spontaneous glomerular disease of *Mpv17* mutant mice, which initially led to death of 90% of the mice at the age of ∼4 months, has become late onset (>1 year of age) in the later breeding, likely due to changes in genetic background [24,25]. When the young *Mpv17* mutant mice that were free of glomerular disease were treated with nephrotoxic serum (NTS), they developed aggravated glomerular podocyte injury, accompanied by an increase of mitochondrial ROS and associated damage in the podocytes, suggesting that MPV17 functions to maintain mitochondrial homeostasis and prevents oxidative stress in podocytes under stresses [25]. We were interested in the role of MPV17 in diabetic kidney disease, and therefore attempted to make *Mpv17* mutant mice diabetic by STZ. To our surprise, *Mpv17* mutant mice were found to be resistant to STZ-induced diabetes.

In the present study, we pursued this observation. We found that the *Mpv17* mutant mice were also resistant to the diabetes induced by insulin 2 mutation (Ins2^Akita^). The *Mpv17* mutant mice of the two diabetic models exhibited ameliorated β-cell apoptosis and loss, demonstrating MPV17 acts to promote β-cell apoptosis, which was further supported by *in vitro* studies with cultured β-cells. Thus, we have identified a novel regulator in β-cell apoptosis.

## Materials and methods

### Mouse care and handling

Mouse handling and treatment were followed the protocols approved by the Animal Care and Use Committee of Mount Sinai School of Medicine (IACUC No. 04-0386-00001-07) and performed in the animal facility located in Annenberg Building of Mount Sinai School of Medicine, New York City, U.S.A. Mouse work was also performed in the animal laboratory located in the building of Department of Comparative Medicine, Jinling hospital, Nanjing, China, and the protocols were approved by the Institutional Animal Care and Use Committee of Jinling Hospital, Nanjing University School of Medicine, China (2015NZGKJ-057). Mice were anesthetized by intraperitoneal injection of sodium pentobarbital (80 mg/kg) in PBS. When the mice were completely anesthetized, perfusion with 4% paraformaldehyde via left ventricle of heart for 5 min was performed, followed by collection of the pancreas for histological analysis. To euthanize, the mice were subjected to CO_2_ inhalation.

### Mpv17 knockout mice

We purchased *Mpv17* global knockout mice on the CFW background (Stock No: 002208) and wild-type CFW mice from the Jackson Laboratory (Bar Harbor, ME, U.S.A.). This *Mpv17* mutant line was generated in a project aiming to randomly disrupt and inactivate genes in mouse embryos by recombinant retrovirus insertion [19]. Mice carrying a single provirus were intercrossed to derive mice homozygous for a given proviral insertion, and the *Mpv17* null mutants (global knockout) were identified in the screening. We used the littermates with different genotypes, including *Mpv17* homozygotes, heterozygotes and wild-type with ages ∼ 8–12-week-old for diabetes induction by streptozotocin.

### STZ treatment for diabetes induction

For multiple low-dose STZ treatment, STZ powder was aliquoted and stored in −20°C until use. At each time of injection, 100 mM sodium citrate buffer (pH 4.2–4.5) was freshly prepared and used to dissolve STZ to a final concentration of 5 mg/ml. The dissolved STZ was immediately injected intraperitonially into the mice within 5 min after dissolution. We used the dose of 50 mg/kg (body weight) of STZ (Sigma-Aldrich,18883-66-4) to treat 8–12-week-old mice by intraperitoneal injection for consecutive 5 days. Blood glucose was monitored once a week and pancreatic tissues were harvested at the time points indicated. For single high-dose STZ treatment, we injected 200 mg/kg (body weight) of the STZ to 8–12-week-old mice intraperitoneally following the method for the multiple low-dose STZ treatment, except that the concentration of STZ solution was adjusted to 20 mg/ml. Blood glucose was measured at the time points indicated.

### Ins2^Akita^-induced diabetes in Mpv17 knockout mice

Ins2^Akita^ mice were purchased from the Jackson Laboratory (Bar Harbor, ME, U.S.A.) (strain: 003548 on the C57B6/J background). This strain carries a spontaneous mutation (Cys96Tyr) in the insulin 2, which causes insulin protein misfolding and toxicity to β cells [16]. Heterozygosity of Ins2^Akita^ mutation is sufficient to induce β-cell death and diabetes. To generate Ins^Akita^-*Mpv17* KO, Ins^Akita^- MPV17 Het, and Ins^Akita^-WT mice, the *Mpv17* mutant allele on CFW background was crossed to Ins^Akita^ mice on C57B6/J background, and the resulting heterozygous F1 mice were further crossed to produce F2 mice. The F2 littermates with the desired genotypes were used each experiment. The detail of breeding strategy is shown in Supplementary Figure S1.

### Blood glucose measurement

Ascensia Coulter (Bayer) was used to measure non-fasting blood glucose level with the blood from tail vein in the morning at 10:00 AM.

### Immunofluorescent staining of insulin, CD3, CD68, and MPV17

Pancreatic tissues from *Mpv17* null mice made diabetic by MLDZ or Ins2^Akita^ mutation were collected after the mice were perfused with 4% paraformaldehyde. Pancreatic tissues were processed for paraffin embedding, followed by 5-μm sectioning. The pancreatic sections were stained with primary antibodies, anti-insulin (Proteintech, 66198), CD3 (Leica Biosystems, NCL-L-CD3-565), CD68 (Leica Biosystems, NCL-L-CD68), or MPV17 (Proteintech, 10310-1-ap), respectively, following the method of immunofluorescent staining previously described [26]. For islet size calculation, 10 islets per animal were assessed from serial sectioning of pancreatic tissues in a blinded manner.

### TUNEL assay

The above pancreatic tissues were also subjected to TUNEL assay to determine apoptosis rates, followed by insulin staining which demarcated islet areas and localized apoptotic nuclei in β-cells. TUNEL assay was performed on the sections using Fluorescein (FITC) TUNEL Cell Apoptosis Detection Kit (G1501–50, Servicebio, Wuhan, China), according to the manual instructions. About 10–20 islets were examined for each mouse. We calculated the number of apoptotic cells in the area of islets in each mouse and thus the apoptotic rate is expressed as # of apoptotic β-cells/total cell number of the islets examined for each mouse.

### SiRNA transfection

MIN6 cells were transfected with scramble or *Mpv17* siRNA using Lipofectamine iMAX (13778030, Invitrogen, Carlsbad, CA, U.S.A.). The sequence of *siMpv17* was as listed: sense-CCACGAAUAGACACGCAUUdTdT, antisense- AAUGCGUGUCUAUUCGUGGdTdT and the final siRNA concentration was 25 nM.

### Palmitic acid and STZ treatment of MIN6 cells

We purchased 10 mM PA in solution complexed with 15% bovine serum albumin (BSA) and BSA control (Kunchuang Biotechnology, Xi’an, China; KC002). We treated MIN6 cells with 0.1- or 0.4-mM palmitate at 37°C in high glucose (25.5 mM) DMEM supplemented with 10% fetal bovine serum, 1% penicillin/streptomycin and 0.05 mmol/L 2-mercaptoethanol, together with control cells treated with the BSA control. For PA treatment, the cells were treated with the PA for additional 24 h after siRNA or scramble transfection. After treatment, the MIN6 cells were lysed in RIPA lysis buffer for insulin quantification, protein concerntration quantification or immunoblotting. 5 mM STZ was used to treat MIN6 similarly.

### Immunoblotting

Protein samples were prepared from MIN6 cells in RIPA lysis buffer containing protease and phosphatase inhibitors (Sigma-Aldrich, PPC1010). Proteins were resolved on Tris/glycine gel and transferred to PVDF membrane (Bio-Rad, Hercules, CA, U.S.A.; 1620177). The following primary antibodies were incubated overnight at 4°C: anti-MPV17 (Proteintech, 10310-1-ap), anti-cleaved caspase-3 (Cell Signaling Technology, 9664s), and anti-GAPDH (Proteintech, 10494-1-ap). Anti-rabbit and anti-mouse secondary antibodies conjugated horseradish peroxidase (Proteintech, SA0001-1, SA0001-2) were used. Specific proteins on the blots were detected using ECL plus substrate (Millipore, WBULS0500).

### ROS measurement

After treatment, MIN6 cells were incubated with 2′,7′-dichlorodihydrofluorescein diacetate (DCFH-DA, Beyotime, S0033S) at 37°C for 1 h in the dark. Then each well was washed three times with serum-free cell culture medium to adequately remove DCFH-DA that did not enter the cells. Absorbance at 488 nm was measured using a microplate reader.

### mtDNA quantification

For mtDNA depletion analysis, mtDNA was extracted from MIN6 as follows: (1) 150 μl of 50 mM NaOH was added to each tube and the cell lysates were boiled at 98°C for 30 min; (2) 15 μl of 1 M Tris-HCl (pH 8.0) was added to each tube and vortexed for 5 s; (3) centrifuged at 6,000 ***g*** for 2 min and the supernatant that containing mtDNA was used for q-PCR analysis.

### Insulin secretion

MIN6 cells in 96-well plate were pre-incubated with DMEM containing 3 mM glucose for 1 h at 37°C in a CO_2_ incubator, followed by incubation with 3 mM glucose DMEM for 4 h, and 100 μl of culture medium was collected from each well for insulin measurement. Then, 16.7 mM glucose DMEM was added to the cells. After incubation for 4 h, 100 μl of medium was collected from each well for insulin measurement. Then, 50 μl of ice-cold 0.1% Triton X-100 was used to lyse the cells for protein content determination using BCA assay. The insulin content in the collected medium and cell lysate was measured using the ELISA kit "Wide range mouse insulin immunoassay kit” (EZassay, MS300), following the manufacturer’s instructions, and normalized by the protein content of the corresponding cell lysates. The insulin content in the cell lysates was also examined using the same ELISA kit.

### Real-time cell analysis

Real-time cell analysis was conducted with the Agilent xCELLigence Real Time Cell Analyzer according to manufacturer’s method. MIN6 cells were seeded into an E-plate 16 PET (Agilent, G009020) and incubated in Agilent xCELLigence Real Time Cell Analyzer after transfection or indicated treatment. The cell states were assessed real time throughout the entire process of treatment and calculated as cell index.

### Cell viability

At the end of treatment, CCK8 solution (1:100, Vazyme, A311-01) was added to each well. The plates were then incubated at 37°C for 1 h in the dark. The absorbance was measured at 450 nm using a microplate reader.

### Statistical analysis

All data are presented as mean ± SD. Comparison between two groups was performed using two-tailed, unpaired Student’s *t*-test, while one-way ANOVA with Dunnett post-hoc test or two-way ANOVA followed by Bonferroni’s post-hoc test was used for the comparison of multiple groups. The level of significance was set at *P*<0.05, 0.01, or 0.001.

## Results

### *Mpv17* knockout mice were resistant to STZ-induced diabetes

**Figure 1:**
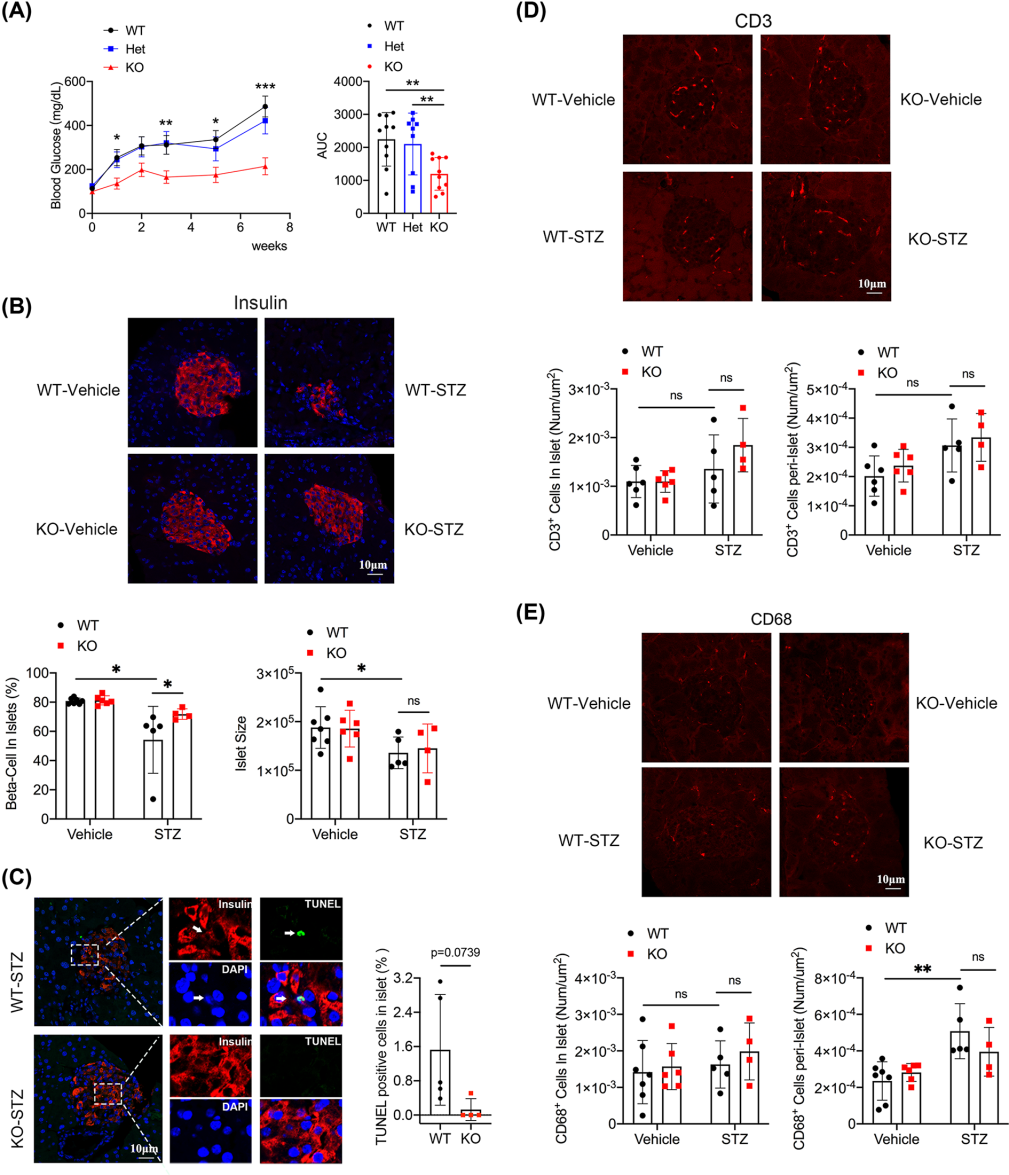
Characterization of mice treated with multiple low-dose STZ (**A**) Time course of blood glucose in the mice of wild-type (WT, *n*=10), heterozygote (Het, *n*=9), and homozygote (KO, *n*=10) of *Mpv17* mutation after STZ treatment. *P<0.05, **P<0.01, ***P<0.000001. (**B**) Fluorescence insulin staining and quantification of β-cell number and islet size of the WT-vehicle (*n*=7), KO-vehicle (*n*=6), WT-STZ (*n*=5), and KO-STZ (*n*=4). (**C**) TUNEL analysis of islets of control and *Mpv17* mutant mice treated with STZ in panel (B). (**D**) CD3^+^ cell infiltration analysis in islets and peri-islets of the mice in panel (B). (**E**) CD68^+^ cell infiltration analysis in islets and peri-islets of the mice in panel (B). All the results in (A) are presented with mean ± SD, and the multiple comparison tests between groups were performed using one-way ANOVA with Dunnett post-hoc test. All the results in (B–E) are presented with mean ± SD and multiple comparison tests between groups were performed using two-way ANOVA followed by Bonferroni’s post-hoc test. *P<0.05, statistically significant; **P<0.01, statistically very significant except for Figure 1A.

To determine whether MPV17 deficiency would aggravate diabetic kidney disease, we treated male *Mpv17* mutant mice aged 8–12 weeks with multiple low-dose STZ (MLDS) for diabetes induction. Unexpectedly, the *Mpv17* mutant mice (KO-STZ) had much lower blood glucose levels than wild-type (WT-STZ) and heterozygous (Het-STZ) mice (Figure 1A). Insulin staining of pancreatic islets showed that STZ treatment caused a reduction of the percentage of β-cells in islets in both KO and WT groups, but the reduction in the KO group was significantly attenuated compared with the WT group (Figure 1B). Meanwhile, the reduction of islet size of *Mpv17* mutant mice was comparable to that of control mice after STZ treatment (Figure 1B). TUNEL assay showed that β-cell apoptosis in the *Mpv17* mutant mice tended to be lower than the control mice (*P*=0.0739) (Figure 1C). Immunofluorescence staining of CD3 showed that the infiltrations of CD3^+^ cells in the islets were comparable between the WT-STZ and KO-STZ groups (*P*=0.5761) (Figure 1D). The peri-islet CD3^+^ cells exhibited similar change as the CD3^+^ in islets among the groups. CD68 staining showed that the infiltrations of CD68^+^ cells in islets of the four groups were at low and comparable levels (Figure 1E). There was no difference in the number of peri-islet CD68^+^ cells between the KO-STZ and WT-STZ mice but the cells were increased in the WT-STZ group versus the WT-vehicle control (*P*=0.0023); in contrast, there was no statistically significant increase in the KO-STZ group versus the KO-vehicle control group (*P*=0.8170) (Figure 1E). As the differences of immune cells infiltration across the groups were subtle, the role of immune cells in β-cell apoptosis, as well as in the differential β-cell loss between *Mpv17* mutant and control mice after STZ treatment require further investigation.

We also performed one high-dose STZ injection, a model in which STZ causes rapid β-cells destruction [27]. The result showed that the *Mpv17* mutant mice (KO) had significantly lower levels of blood glucose than the wild-type controls (WT) (Supplementary Figure S2).

### *Mpv17* knockout mice were resistant to Ins2^Akita^-induced diabetes

**Figure 2:**
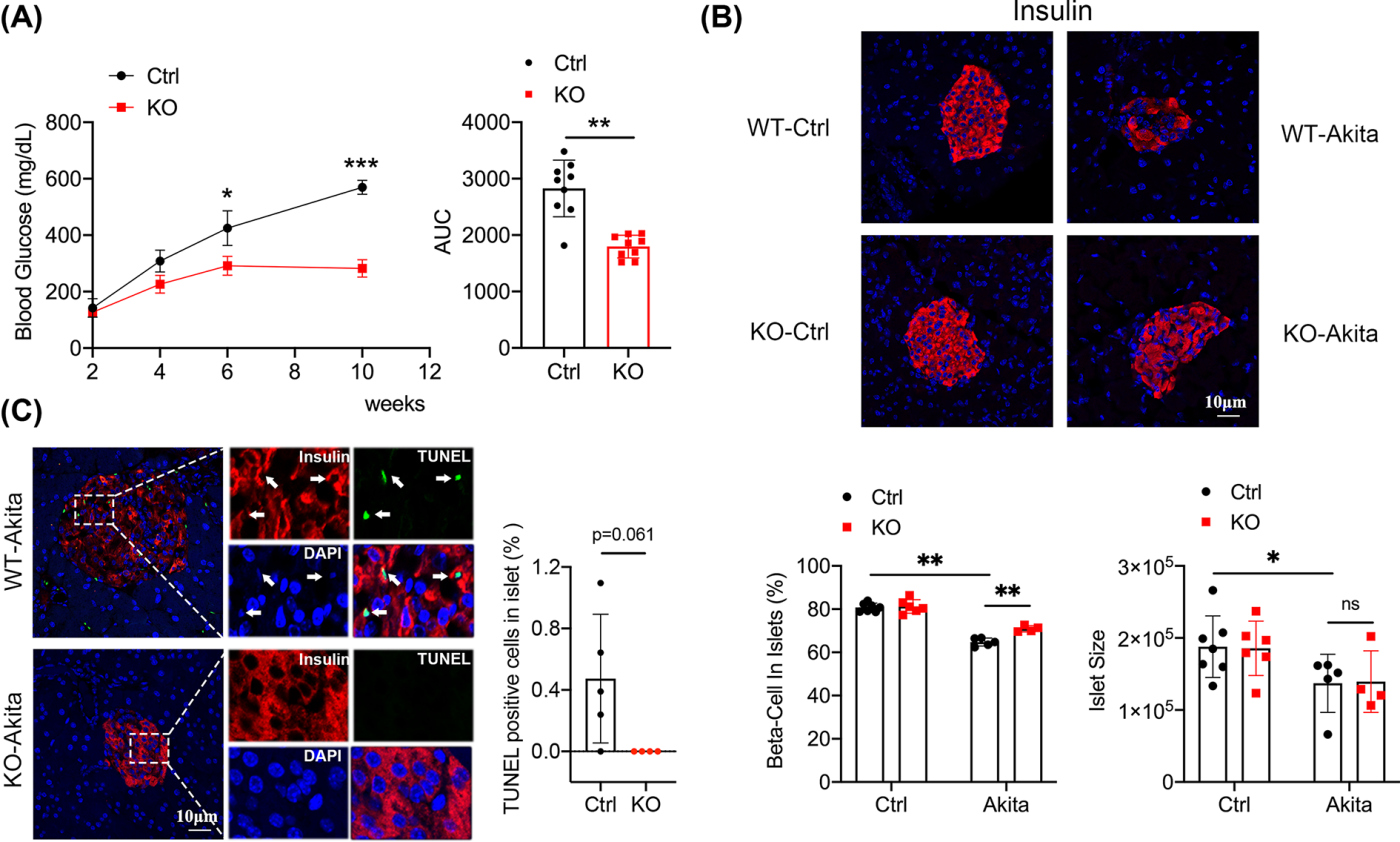
Characterization of *Mpv17* wild-type and mutant mice carrying Ins2^Akita^ mutation (**A**) The time course of blood glucose levels in the mice and AUC comparison. Ctrl: wild-type control (*n*=9), KO: *Mpv17* mutant (*n*=9). *P<0.05, **P<0.0001, ***P<0.000001, statistically significant. (**B**) Fluorescence staining of insulin in the mice as indicated, and quantifications of β-cell number and islet size of the mice. WT-vehicle (*n*=7), wild-type-Akita (*n*=5), KO-vehicle (*n*=6), KO-Akita (*n*=4). *P<0.05, **P<0.01, statistically significant. (**C**) TUNEL assay and insulin staining showing apoptosis in β-cells of the WT-Akita and Mpv17 KO-Akita mice in panel (B). Quantification of the results is showed on the right; *P=0.061. All the results are presented with mean ± SD. The comparisons between two groups were performed using two-tailed, unpaired Student’s *t*-test (**A,C**), and the multiple comparison tests between groups were performed using two-way ANOVA followed by Bonferroni’s post-hoc test (B).

We next tested the *Mpv17* mutant mice in Ins2^Akita^-induced diabetogenesis. *Mpv17* mutant allele was crossed to the Ins2^Akita^ allele and the *Mpv17*^−/−^, Ins2^Akita^ mice (KO-Akita) were obtained, and KO-Akita mice had significantly lower levels of blood glucose than Ins2^Akita^ control mice (WT-Akita) (Figure 2A). Consistently, β-cell percentage in islets of the KO-Akita mice was higher than that of control mice (Figure 2B), as expected. A lower level of apoptosis was found in the KO-Akita mice compared with the control mice with the *P*=0.061 (Figure 2C). CD3^+^ cells infiltrations in the two groups of Akita mice (WT-Akita and KO-Akita) were significantly increased compared with the non-Akita control groups; however, the increases of CD3^+^ cells infiltration were comparable between the KO-Akita and WT-Akita (*P*>0.9999) (Supplementary Figure S3A). As for the infiltration of CD68^+^ cells, there were no statistical differences between the four groups (Supplementary Figure S3B). The body weight of the KO-Akita mice was indistinguishable from that of the WT-Akita mice before the difference in blood glucose took place (Supplementary Figure S4).

### β-Cell apoptosis correlated with β-cell number, insulin content and blood glucose in the STZ and Ins2^Akita^-induced diabetes

We performed correlation analyses between β-cell apoptosis and β-cell number, blood glucose level, immune cell infiltration in STZ and Akita models (Supplementary Figures S5 and 6, respectively), and found that β-cell apoptosis was negatively correlated with β-cell number, but positively correlated with blood glucose, suggesting that β-cell apoptosis caused β-cell loss and the resulting hyperglycemia. β-cell apoptosis had no or weak correlation with immune cell infiltration. Together with the observation that β-cell apoptosis was reduced in *Mpv17* mutant mice both in STZ and Akita models, these results of correlation study support that MPV17 may promote β-cell apoptosis and its deficiency ameliorates β-cell loss and diabetes.

### Mpv17 is expressed in β-cells

**Figure 3:**
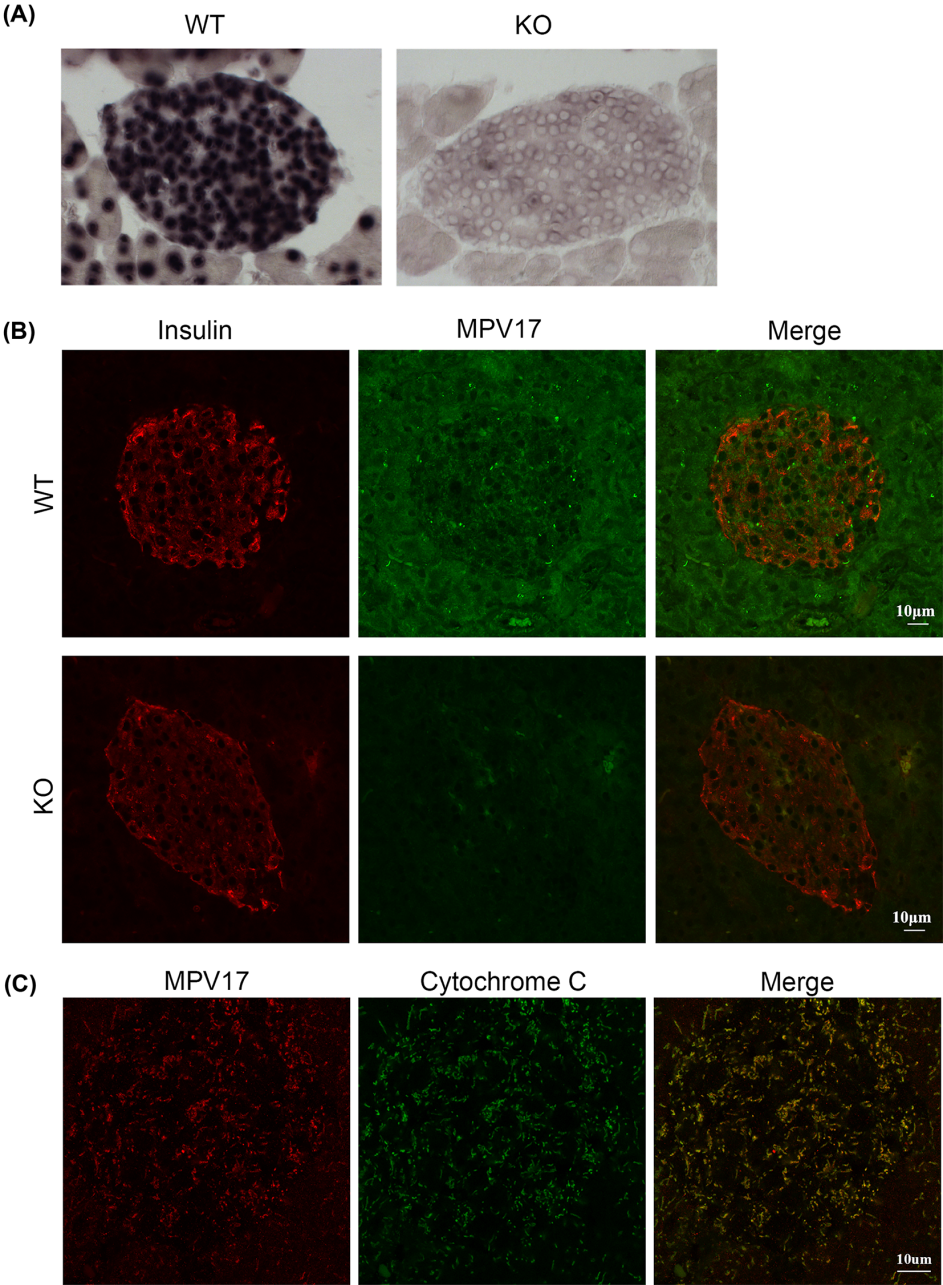
MPV17 expression in β-cells (**A**) RNA *in situ* hybridization of *Mpv17* in pancreatic islets of wild-type and mutant mice. (**B**) Immunofluorescent co-staining of MPV17 and insulin in pancreatic islets of wild-type and mutant mice. (**C**) Confocal microscopy images of immunofluorescent co-staining show that MPV17 protein is colocalized with the mitochondrial marker cytochrome *c*.

To prove that MPV17 deficiency-conferred resistance to diabetes is β-cell autonomous, we examined whether MPV17 is expressed in β-cells. RNA *in situ* hybridization showed abundant *Mpv17* mRNA in all cells in islet of wild-type (WT) but not *Mpv17* mutant mice (KO) (Figure 3A). Immunofluorescent co-staining with insulin confirmed the presence of MPV17 protein in the islet cells of WT but not KO mice (Figure 3B). We also found that MPV17 protein colocalized with cytochrome *c* as shown by confocal microscopy (Figure 3C), consistent with previous reports that MPV17 is a mitochondrial protein [20].

### *Mpv17* knockdown alleviated STZ-induced apoptosis in cultured β-cells

**Figure 4:**
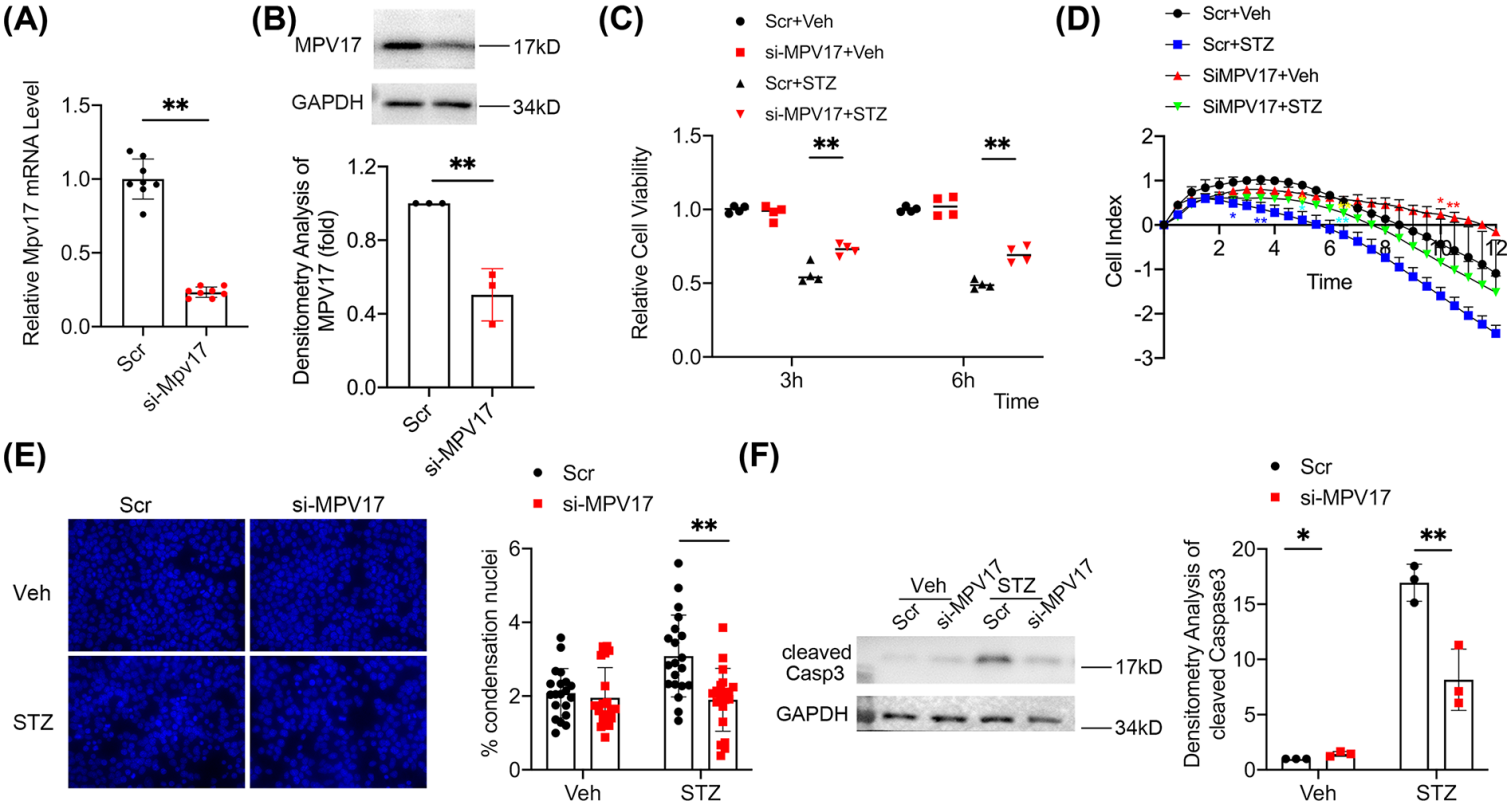
*Mpv17* knockdown in cultured β-cell, MIN6, protected the cells against STZ-induced injury and apoptosis (**A**) qPCR and immunoblotting analysis of MPV17 levels in the Mpv17 siRNA-and scramble-treated MIN6 cells. (**B**) Immunoblotting showing MPV17 protein knockdown efficiency by the si-*Mpv17*. (**C**) CCK8 assay showed attenuated decrease of cell viability of the Mpv17 knockdown cells. (**D**) Cell real-time assay showed that *Mpv17* knockdown attenuated the decrease of cell viability in the treatment of STZ. *P<0.05, statistically significant; **P<0.01, statistically very significant. The blue stars refer to the Scr+Veh vs. Scr+STZ, yellow stars to si-*Mpv17*+Veh vs. si-*Mpv17*+STZ, turquoise stars to Scr+STZ vs. si-*Mpv17*+STZ, and red stars to Scr+Veh vs. si-*Mpv17*+Veh. (**E**) Condensation nuclei counting showed that STZ-induced MIN6 apoptosis was alleviated by *Mpv17* knockdown. (**F**) Immunoblotting showed the cleaved caspase 3 was increased by STZ treatment, which was attenuated in the cells treated with si-*Mpv17*. Quantification of the result is shown on the right. The quantification result in each panel is presented with mean ± SD of the data from three independent experiments except (E) in which the quantification is presented with mean ± SD of the counts of condensation nuclei from 20 random high-power fields. The comparisons between two groups were performed using two-tailed, unpaired Student’s *t*-test (A,B) and the multiple comparison tests between groups were performed using two-way ANOVA followed by Bonferroni’s post-hoc test (C–F). *P<0.05, statistically significant; **P<0.01, statistically very significant.

We performed *in vitro* study to investigate the role of MPV17 in β-cell injury. *Mpv17* was knocked down by siRNA in β-cell line, MIN6. Both qPCR and Western blotting showed a significant reduction of MPV17 in the cells of *Mpv17*-knockdown group (Figure 4A, B). After the treatment with STZ, the *Mpv17*-knockdown cells exhibited better morphology compared with the scramble controls (data not shown). CCK8 cell viability assay and cell real-time assay both showed improved cell viability of the *Mpv17* knockdown cells versus the control cells in the treatment of STZ (Figure 4C,D). Condensation nuclei counting showed a lower level of apoptosis in the *Mpv17*-knockdown cells compared with the scramble controls (Figure 4E), accompanied by a lower level of caspase 3 activation (Figure 4F), demonstrating that MPV17 promotes STZ-induced β-cell apoptosis.

### *Mpv17* knockdown alleviated palmitic acid-induced apoptosis in cultured β-cells

**Figure 5:**
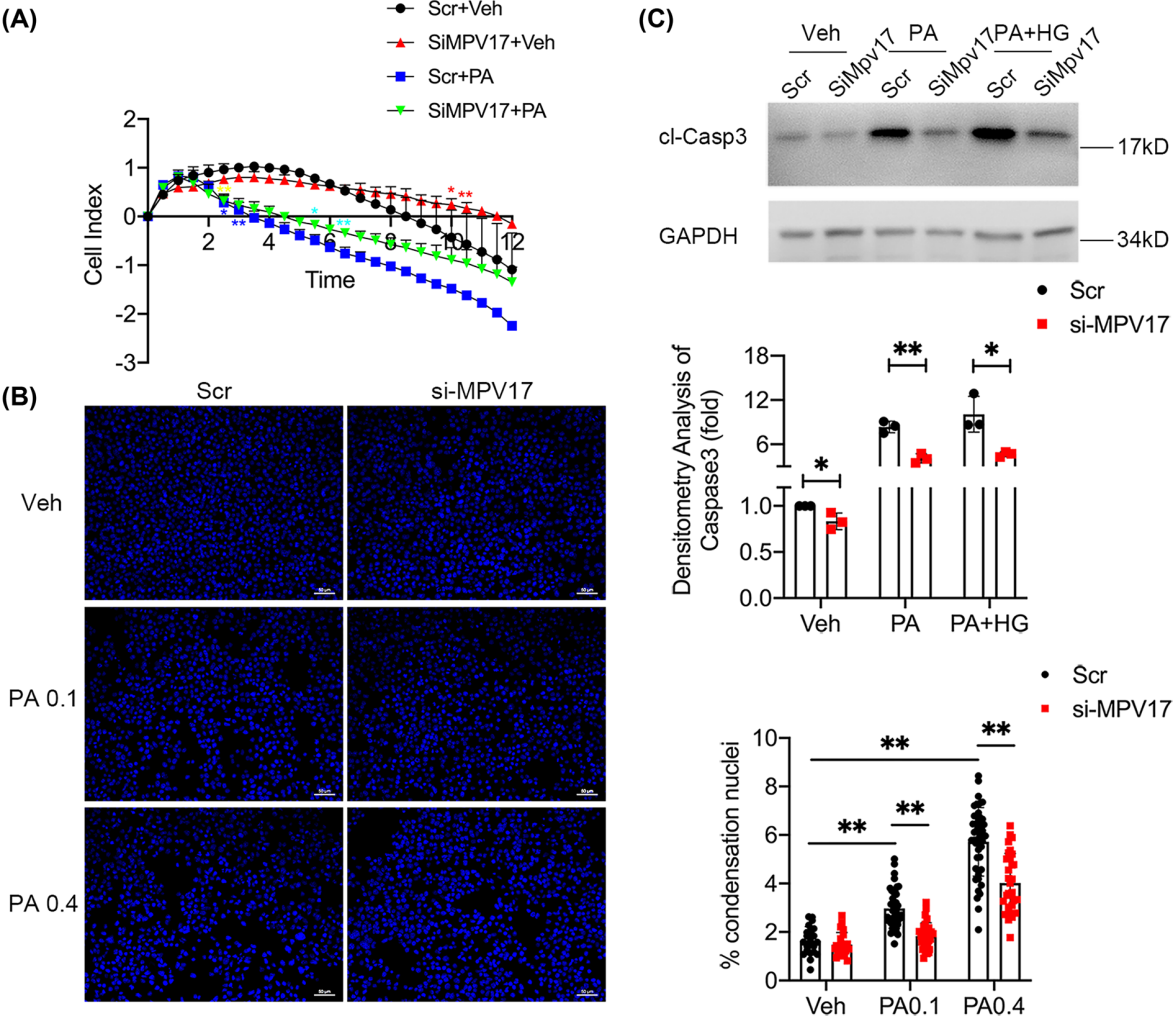
*Mpv17* knockdown in cultured β-cell, MIN6, protected against PA-induced injury and apoptosis (**A**) Cell real-time assay showed attenuated decrease in viability of the cells treated with si-*Mpv17* followed by PA. The result is presented with mean ± SD of the replicate wells. *P<0.05, statistically significant; **P<0.01, statistically very significant. The blue stars refer to the comparison of Scr+Veh vs. Scr+PA, yellow stars to si-*Mpv17*+Veh vs. si-*Mpv17*+PA, turquoise stars to Scr+PA vs. si-*Mpv17*+PA, and red stars to Scr+Veh vs. si-*Mpv17*+Veh. (**B**) Condensation nuclei counting showed attenuated apoptosis in the cells with *Mpv17* knockdown in panel (A) compared with the control. The result is the mean ± SD of the counts of condensation nuclei from 20 random fields. (**C**) Immunoblotting shows that cleaved caspase 3 was reduced in the cells with *Mpv17* knockdown in the treatment of PA. The results are presented with mean ± SD from three independent experiments. The comparisons between groups were performed using two-way ANOVA followed by Bonferroni’s post-hoc test (**B**). *P<0.05, statistically significant; **P<0.01, statistically very significant.

As shown above, *Mpv17* deficiency also attenuated Ins2^Akita^-induced β-cell loss and diabetes, suggesting that MPV17 promotes ER stress-induced β-cell injury. We used palmitic acid (PA), a model featured ER stress as in the β-cells with Ins2^Akita^, to determine whether MPV17 deficiency attenuates ER stress-induced β-cell apoptosis. We found that *Mpv17* knockdown protected the MIN6 cells against PA as evidenced by an attenuated decrease in cell viability in the si-*Mpv17* treated cells compared with the scramble control (Figure 5A). Nuclear condensation assay also showed mitigated apoptosis in the si-*Mpv17* treated cells (Figure 5B). Consistently, si-*Mpv17*-treated cells had a much lower level of cleaved caspase 3 (Figure 5C).

### Mpv17 did not affect the ROS and mtDNA levels in β-cells under STZ or PA treatment

**Figure 6:**
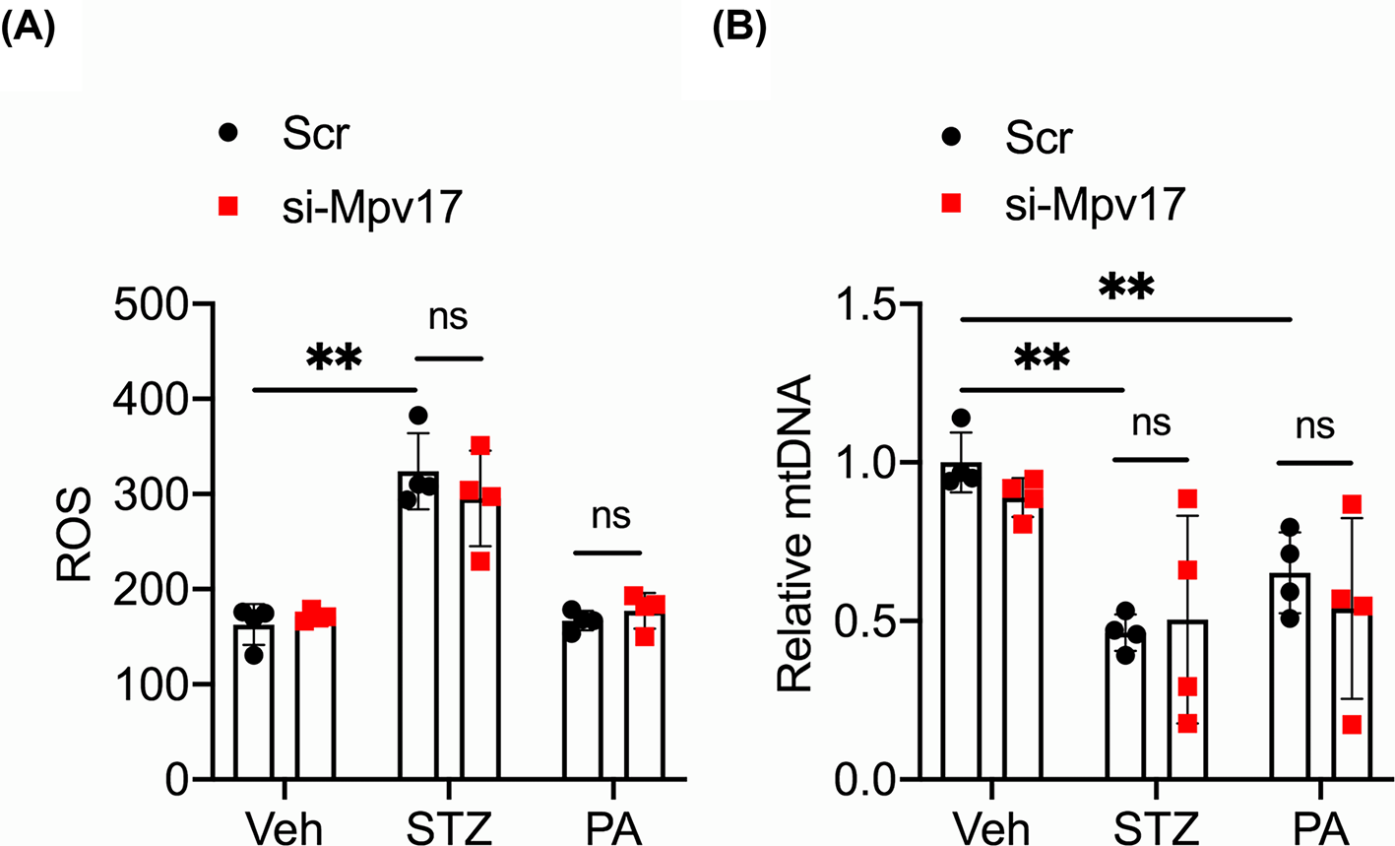
*Mpv17* deficiency did not affect ROS metabolism and mtDNA stability in cultured MIN6 cells. Mpv17 deficiency did not affect ROS metabolism ( **A**) and mtDNA stability (**B**) in cultured MIN6 cells. All the results are presented with mean ± SD. Comparisons between groups were performed using two-way ANOVA followed by Bonferroni’ post-hoc test; ns: no significant difference.

MPV17 resides in the inner membrane of a mitochondrion and its deficiency has been shown to cause mitochondrial ROS increase and mtDNA depletion in several cell types [20,25], which facilitate apoptosis of the cells [28]. We found that the cells treated with si-*Mpv17* had comparable ROS and mtDNA levels with scramble control cells under STZ or PA treatment (Figure 6A,B), suggesting that MPV17 promotes β-cell apoptosis downstream of ROS increase and mtDNA depletion.

### Mpv17 knockdown did not affect insulin secretion

Glucose stimulated insulin secretion is a means of functional assessment of β-cells. To determine whether MPV17 is involved in insulin secretion, MIN6 cells were treated with scramble and si-*Mpv17*, respectively, and stimulated with low glucose (3 mM) and then high glucose (16.7 mM) for 4 h. The insulin contents in supernatant of the medium and cell lysate were measured by ELISA. The results showed that the si-*Mpv17* cells had a similar level of insulin secretion to that of scramble control cells after high glucose stimulation (Supplementary Figure S7A). Meanwhile, si-*Mpv17* treated cells had a similar level of intracellular insulin as the control cells (Supplementary Figure S7B), suggesting that MPV17 deficiency does not affect insulin expression in β-cells. These results further support that MPV17 deficiency confers diabetes resistance by preventing β-cell loss.

## Discussion

In the present study, we show that MPV17 is expressed in β cells and that *Mpv17* deficiency confers resistance to apoptosis in the two mouse models of diabetes, STZ and Ins2^Akita^, indicating that MPV17 acts in β-cells to promote apoptosis and facilitate the development of diabetes. β-Cell autonomous action of MPV17 was also demonstrated by the one high-dose STZ model. The proapoptotic effect of MPV17 was confirmed in cultured β-cells. Thus, this study has identified a novel regulator of β-cell apoptosis, potentially facilitating the development of intervention for Type 1 diabetes and some monogenic forms of diabetes.

MPV17-conferred protection on β-cells occurs in two distinct diabetic mouse models, STZ and Ins2^Akita^, suggesting that MPV17 may be involved in a common event in STZ- and Ins2^Akita^-induced diabetes. This common event may not be immune cell infiltration and attack, as the differences in T-cell infiltration levels between the *Mpv17* mutant and control groups in the two models were not significant. In fact, Ins2^Akita^ model is known to be independent of autoimmunity and T-cell infiltration [16,17]; and the immune defective SCID mice, which lack immune cell infiltration in islets, are similarly sensitive to STZ for diabetic development [12]. β-Cell loss that is independent of immune attack has also been observed in other types of diabetes [8,9]. As β-cell apoptosis is the common event in the MLDS and Ins2^Akita^ models, and β-cell apoptosis was attenuated in the *Mpv17* knockout mice in both diabetic models, we suggest that MPV17 facilitates diabetogenesis mechanistically by promoting β-cell apoptosis. We have further confirmed MPV17 pro-apoptotic effect in cultured β-cells treated with STZ, the *in vitro* model of STZ-induced β-cell death, and PA, a model that shares feature of ER stress induction with the Ins2^Akita^ in β-cells, respectively. Thus, for the first time we have found the deleterious effect of MPV17 in contrast with its protective effect [19–25].

MPV17 deficiency causes mtDNA depletion and ROS elevation, leading to apoptosis in hepatocytes [20], glomerular podocytes [25,29], and neuronal cells [30]. MPV17 deficiency is also deleterious in cardiomyocytes and aggravates ischemia–reperfusion injury [31]. In contrast, in β-cells MPV17 deficiency is protective against STZ-, PA-, and Ins2^Akita^-induced apoptosis. As MPV17 knockdown in MIN6 cells did not affect mtDNA and ROS dynamics, MPV17 may act downstream of mtDNA depletion and ROS increase in β-cells, which is in contrast with the cell types mentioned above [25,29]. It will be important to elucidate the mechanism by which MPV17 mediates β-cell apoptosis. However, this task would be challenging given that MPV17 has not been extensively studied and the knowledge of its functions and mechanisms is limited.

Although the role of MPV17 in β-cell apoptosis has been well demonstrated in the Ins2^Akita^ mice, one of the mouse models of human monogenic diabetes, in the present study, its role in Type 1 diabetes needs to be further confirmed because STZ model does not adequately represent the autoimmune etiology of Type 1 diabetes. NOD strain is considered the best mouse model for human autoimmune T1DM because it shares many similarities in etiology including pancreas-specific auto-antibodies, autoreactive T cells and islet infiltration, and even some genetic loci that predisposes to autoimmunity [32,33]. It is important to introduce *Mpv17* mutation to NOD mice to confirm the role of MPV17 in β-cell apoptosis of Type 1 diabetes. For Type 2 diabetes, the role of MPV17 in its development is not known because the contribution of β-cell death to Type 2 diabetes development remains a controversial debate topic [3,34,35].

It has been shown that β-cell dedifferentiation and dysfunction play important roles in diabetes development. Talchai et al. and Cinti et al. showed that β-cell dedifferentiation is a critical mechanism of diabetic β-cell failure in Type 2 diabetes development [4,5]. However, Butler et al. did not observe β-cell dedifferentiation in the islets of the Type 2 diabetic patients enrolled in the study [35]. Hence, it seems that β-cell dedifferentiation, dysfunction, and death in Type 2 diabetic mice and patients may be dependent on Type 2 diabetes subtypes. Interestingly, in the present study we observed in the TUNEL assay that insulin intensity around the apoptotic nuclei was diminished (Figures 1C and 2C). Whether this phenomenon is caused by β-cell dedifferentiation prior to apoptosis, or by other events, e.g., hypersecretion of insulin before death or diffusion of insulin from the β-cell due to impaired membrane integrity requires further investigation. Associated with this issue, we observed that MPV17 did not affect insulin secretion and expression based on the assay with MIN6 cells (Supplementary Figure S6). It would be interesting to investigate the role of MPV17 in β-cell dedifferentiation and dysfunction in various types of diabetes.

There are several limitations in the present study. First, this study lacked metabolic data of the mice. This is important given that our study used global knockout of *Mpv17* that may affect hepatic or other tissues’ glucose production thereby contributing to the observed attenuation of hyperglycemia in the knockout mice. Although the blood glucose of *Mpv17* knockout mice was normal, the possibility in diabetic induction could not be precluded. In this regard, generation of conditional *Mpv17* knockout mice would be necessary. Second, the number of animals used in each experiment was low. Although the low number issue was partly addressed by using multiple diabetic models that consistently showed the diabetic resistance conferred by *Mpv17* deficiency, increasing the number of animals would certainly give rise to more reliable results. Third, *Mpv17* global, but not β-cell specific, knockout mice were used in the study. It is necessary to generate *Mpv17* conditional knockout mice to further confirm the results in the present study. In addition, β-cell and podocyte specific knockout of *Mpv17* would provide tools to address the opposite effects of MPV17 in the two cell types, providing insights into the mechanisms underlying not only diabetes but also diabetic kidney disease. In fact, determining the role of MPV17 in podocyte injury in diabetes was our original aim of study. This is an important research topic because diabetic kidney disease is one of the major diabetic complications, affecting 40% of diabetic patients [36,37].

## Clinical perspectives

- β-Cell death contributes to the development and progression of Type 1 diabetes as well as some monogenic forms of diabetes, while the mechanism of β-cell death remains incompletely understood. By accident, we found that *Mpv17* knockout mice are resistant to STZ-induced diabetes. We aim to determine the role of MPV17 in β-cell death, hopefully identifying a novel regulator of β-cell death.
- *Mpv17* knockout mice were resistant to not only STZ-induced diabetes but also proinsulin mutation (Ins2^Akita^) induced diabetes. In both diabetes models, *Mpv17* knockout mice exhibited lower levels of blood glucose, β-cell loss, and apoptosis. In cultured β-cells, *Mpv17* knockdown resulted in attenuated caspase 3 activation and apoptosis in the treatment of STZ or palmitic acid. Therefore, MPV17 promotes β-cell apoptosis in diabetes development.
- Identification of MPV17 as a novel regulator of β-cell death has provided a potential target for treatment of Type 1 diabetes and some monogenic forms of diabetes.

## Supplementary Material

Supplementary Figures S1-S7

## Data Availability

The data underlying this article will be shared on reasonable request to the corresponding authors.

## Abbreviations

BSA: bovine serum albumin
PA: palmitic acid
DCFH-DA: 2′,7′-dichlorodihydrofluorescein diacetate
ER: endoplasmic reticulum
ROS: reactive oxygen species
STZ: streptozotocin
mtDNA: mitochondrial DNA

## References

[1] Katsarou A., Gudbjörnsdottir S., Rawshani A., Dabelea D., Bonifacio E., Anderson B. et al. (2017) Type 1 diabetes mellitus. Nat. Rev. Dis. Primers 3, 17016 10.1038/nrdp.2017.16 28358037

[2] Roep B.O., Thomaidou S., van Tienhoven R. and Zaldumbide A. (2021) Type 1 diabetes mellitus as a disease of the β-cell (do not blame the immune system?) Nat. Rev. Endocrinol. 17, 150–161, PMCID: PMC7722981 10.1038/s41574-020-00443-4 33293704 PMC7722981

[3] Montane J., Cadavez L. and Novials A. (2014) Stress and the inflammatory process: a major cause of pancreatic cell death in type 2 diabetes. Diabetes Metab. Syndr Obes. 7, 25–34, PMCID: PMC3917922 24520198 10.2147/DMSO.S37649 PMC3917922

[4] Talchai C., Xuan S., Lin H.V., Sussel L. and Accili D. (2012) Pancreatic β cell dedifferentiation as a mechanism of diabetic β cell failure. Cell 150, 1223–1234, PMCID: PMC3445031 10.1016/j.cell.2012.07.029 22980982 PMC3445031

[5] Cinti F., Bouchi R., Kim-Muller J.Y., Ohmura Y., Sandoval P.R., Masini M. et al. (2016) Evidence of β-cell dedifferentiation in human type 2 diabetes. J. Clin. Endocrinol. Metab. 101, 1044–1054, PMCID: PMC4803182 10.1210/jc.2015-2860 26713822 PMC4803182

[6] Yamada T., Ishihara H., Tamura A., Takahashi R., Yamaguchi S., Takei D. et al. (2006) WFS1-deficiency increases endoplasmic reticulum stress, impairs cell cycle progression and triggers the apoptotic pathway specifically in pancreatic beta-cells. Hum. Mol. Genet. 15, 1600–1609 10.1093/hmg/ddl081 16571599

[7] Colombo C., Porzio O., Liu M., Massa O., Vasta M., Salardi S. et al. (2008) Early onset diabetes study group of the italian society of pediatric endocrinology and diabetes (SIEDP). Seven mutations in the human insulin gene linked to permanent neonatal/infancy-onset diabetes mellitus. J. Clin. Invest. 118, 2148–2156, PMCID: PMC2350430 18451997 10.1172/JCI33777 PMC2350430

[8] Liu M., Huang Y., Xu X., Li X., Alam M., Arunagiri A. et al. (2021) Normal and defective pathways in biogenesis and maintenance of the insulin storage pool. J. Clin. Invest. 131, e142240, PMCID: PMC7810482 10.1172/JCI142240 33463547 PMC7810482

[9] Wilcox N.S., Rui J., Hebrok M. and Herold K.C. (2016) Life and death of β cells in Type 1 diabetes: A comprehensive review. J. Autoimmun. 71, 51–58, PMCID: PMC4903951 10.1016/j.jaut.2016.02.001 27017348 PMC4903951

[10] Cole J.B. and Florez J.C. (2020) Genetics of diabetes mellitus and diabetes complications. Nat. Rev. Nephrol. 16, 377–390 10.1038/s41581-020-0278-5 32398868 PMC9639302

[11] Predieri B., Bruzzi P., Bigi E., Ciancia S., Madeo S.F., Lucaccioni L. et al. (2020) Endocrine Disrupting Chemicals and Type 1 Diabetes. Int. J. Mol. Sci. 21, 2937, PMCID: PMC7215452 10.3390/ijms21082937 32331412 PMC7215452

[12] Reddy S., Wu D. and Elliott R.B. (1995) Low dose streptozotocin causes diabetes in severe combined immunodeficient (SCID) mice without immune cell infiltration of the pancreatic islets. Autoimmunity 20, 83–92 10.3109/08916939509001931 7578872

[13] Deeds M.C., Anderson J.M., Armstrong A.S., Gastineau D.A., Hiddinga H.J., Jahangir A. et al. (2011) Single dose streptozotocin-induced diabetes: considerations for study design in islet transplantation models. Lab. Anim. 45, 131–140, PMCID: PMC3917305 10.1258/la.2010.010090 21478271 PMC3917305

[14] Watanabe A., Nishijima K., Zhao S., Zhao Y., Tanaka Y., Takemoto H. et al. (2012) Quantitative determination of apoptosis of pancreatic β-cells in a murine model of type 1 diabetes mellitus. J. Nucl. Med. 53, 1585–1591 10.2967/jnumed.111.102459 22930815

[15] Liu M., Hodish I., Haataja L., Lara-Lemus R., Rajpal G., Wright J. et al. (2010) Proinsulin misfolding and diabetes: mutant INS gene-induced diabetes of youth. Trends Endocrinol. Metab. 21, 652–659, PMCID: PMC2967602 10.1016/j.tem.2010.07.001 20724178 PMC2967602

[16] Yoshioka M., Kayo T., Ikeda T. and Koizumi A. (1997) A novel locus, Mody4, distal to D7Mit189 on chromosome 7 determines early-onset NIDDM in nonobese C57BL/6 (Akita) mutant mice. Diabetes 46, 887–894 10.2337/diab.46.5.887 9133560

[17] Oyadomari S., Koizumi A., Takeda K., Gotoh T., Akira S., Araki E. et al. (2002) Targeted disruption of the Chop gene delays endoplasmic reticulum stress-mediated diabetes. J. Clin. Invest. 109, 525–532, PMCID: PMC150879 10.1172/JCI0214550 11854325 PMC150879

[18] Yong J., Johnson J.D., Arvan P., Han J. and Kaufman R.J. (2021) Therapeutic opportunities for pancreatic β-cell ER stress in diabetes mellitus. Nat. Rev. Endocrinol. 17, 455–467, PMCID: PMC8765009 10.1038/s41574-021-00510-4 34163039 PMC8765009

[19] Weiher H., Noda T., Gray D.A., Sharpe A.H. and Jaenisch R. (1990) Transgenic mouse model of kidney disease: insertional inactivation of ubiquitously expressed gene leads to nephrotic syndrome. Cell 62, 425–434 10.1016/0092-8674(90)90008-3 1696177

[20] Spinazzola A., Viscomi C., Fernandez-Vizarra E., Carrara F., D'Adamo P., Calvo S. et al. (2006) MPV17 encodes an inner mitochondrial membrane protein and is mutated in infantile hepatic mitochondrial DNA depletion. Nat. Genet. 38, 570–575 10.1038/ng1765 16582910

[21] El-Hattab A.W., Wang J., Dai H., Almannai M., Scaglia F., Craigen W.J. et al. (2012) MPV17-related mitochondrial DNA maintenance defect. In GeneReviews® [Internet](Adam M.P., Mirzaa G.M., Pagon R.A., Wallace S.E., Bean L.J.H., Gripp K.W. et al., eds), pp. 1993–2022, University of Washington, Seattle, Seattle (WA), [updated 2018 May 17]. 22593919

[22] Zwacka R.M., Reuter A., Pfaff E., Moll J., Gorgas K., Karasawa M. et al. (1994) The glomerulosclerosis gene Mpv17 encodes a peroxisomal protein producing reactive oxygen species. EMBO J. 13, 5129–5134, PMCID: PMC395460 10.1002/j.1460-2075.1994.tb06842.x 7957077 PMC395460

[23] Binder C.J., Weiher H., Exner M. and Kerjaschki D. (1999) Glomerular overproduction of oxygen radicals in Mpv17 gene-inactivated mice causes podocyte foot process flattening and proteinuria: A model of steroid-resistant nephrosis sensitive to radical scavenger therapy. Am. J. Pathol. 154, 1067–1075, PMCID: PMC1866567 10.1016/S0002-9440(10)65359-X 10233845 PMC1866567

[24] O'Bryan T., Weiher H., Rennke H.G., Kren S. and Hostetter T.H. (2000) Course of renal injury in the Mpv17-deficient transgenic mouse. J. Am. Soc. Nephrol. 11, 1067–1074 10.1681/ASN.V1161067 10820170

[25] Casalena G., Krick S., Daehn I., Yu L., Ju W., Shi S. et al. (2014) Mpv17 in mitochondria protects podocytes against mitochondrial dysfunction and apoptosis in vivo and in vitro. Am. J. Physiol. Renal. Physiol. 306, F1372–F1380, PMCID: PMC4042102 10.1152/ajprenal.00608.2013 24598802 PMC4042102

[26] Lu Y., Ye Y., Yang Q. and Shi S. (2017) Single-cell RNA-sequence analysis of mouse glomerular mesangial cells uncovers mesangial cell essential genes. Kidney Int. 92, 504–513 10.1016/j.kint.2017.01.016 28320530

[27] Lenzen S. (2008) The mechanisms of alloxan- and streptozotocin-induced diabetes. Diabetologia 51, 216–226 10.1007/s00125-007-0886-7 18087688

[28] Dirks A.J., Hofer T., Marzetti E., Pahor M. and Leeuwenburgh C. (2006) Mitochondrial DNA mutations, energy metabolism and apoptosis in aging muscle. Ageing Res. Rev. 5, 179–195 10.1016/j.arr.2006.03.002 16647308

[29] Luna-Sanchez M., Benincá C., Cerutti R., Brea-Calvo G., Yeates A., Scorrano L. et al. (2020) Opa1 overexpression protects from early-onset Mpv17-/–related mouse kidney disease. Mol. Ther. 28, 1918–1930, PMCID: PMC7403474 10.1016/j.ymthe.2020.06.010 32562616 PMC7403474

[30] Li A., Li L., Sun X., Ni Y., Chen X., Guo A. et al. (2015) Increased expression of mitochondrial inner-membrane protein Mpv17 after intracerebral hemorrhage in adult rats. Neurochem. Res. 40, 1620–1630 10.1007/s11064-015-1644-8 26123482

[31] Madungwe N.B., Feng Y., Imam Aliagan A., Tombo N., Kaya F. and Bopassa J.C. (2020) Inner mitochondrial membrane protein MPV17 mutant mice display increased myocardial injury after ischemia/reperfusion. Am. J. Transl. Res. 12, 3412–3428, PMCID: PMC7407695 32774709 PMC7407695

[32] Padgett L.E., Broniowska K.A., Hansen P.A., Corbett J.A. and Tse H.M. (2013) The role of reactive oxygen species and proinflammatory cytokines in type 1 diabetes pathogenesis. Ann. N. Y. Acad. Sci. 1281, 16–35, PMCID: PMC3715103 10.1111/j.1749-6632.2012.06826.x 23323860 PMC3715103

[33] Pearson J.A., Wong F.S. and Wen L. (2016) The importance of the non obese diabetic (NOD) mouse model in autoimmune diabetes. J. Autoimmun. 66, 76–88, PMCID: PMC4765310 10.1016/j.jaut.2015.08.019 26403950 PMC4765310

[34] Tornovsky B.S., Dadon D., Ziv O., Tzipilevich E., Kadosh T., Schyr B.H.R. et al. (2014) Type 2 diabetes and congenital hyperinsulinism cause DNA double-strand breaks and p53 activity in beta cells. Cell Metab. 19, 109–121 10.1016/j.cmet.2013.11.007 24332968

[35] Butler A.E., Dhawan S., Hoang J., Cory M., Zeng K., Fritsch H. et al. (2016) Beta-cell deficit in obese type 2 diabetes, a minor role of beta-cell dedifferentiation and degranulation. J. Clin. Endocrinol. Metab. 101, 523–532, PMCID: PMC4880126 10.1210/jc.2015-3566 26700560 PMC4880126

[36] Hou J.H., Zhu H.X., Zhou M.L., Le W.B., Zeng C.H., Liang S.S. et al. (2018) Changes in the spectrum of kidney diseases: an analysis of 40,759 biopsy-proven cases from 2003 to 2014 in China. Kidney Dis. (Basel.) 4, 10–19, PMCID: PMC5848489 10.1159/000484717 29594138 PMC5848489

[37] Satirapoj B. and Adler S.G. (2015) Prevalence and management of diabetic nephropathy in western countries. Kidney Dis. (Basel.) 1, 61–70, PMCID: PMC4934803 10.1159/000382028 27536666 PMC4934803

