# Supplementary material for: Mitochondrial protein MPV17 promotes β-cell apoptosis in diabetogenesis
Source: Clin Sci (Lond). 2023 Aug 10;137(15):1195–208. doi: 10.1042/CS20230164 (PMC10415165; doi:10.1042/CS20230164)
Supplement: Supplementary Figures S1-S7 [file CS-2023-0164_supp.pdf]

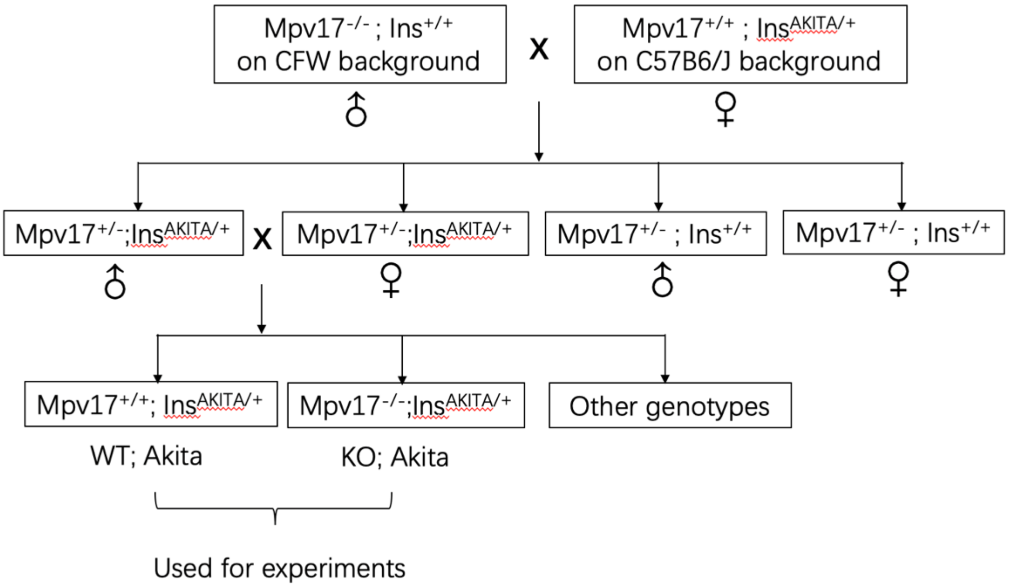

Supplemental Figure 1. Breeding strategy for production of Mpv17 wild-type and knockout mice carrying Ins^{Akita}.

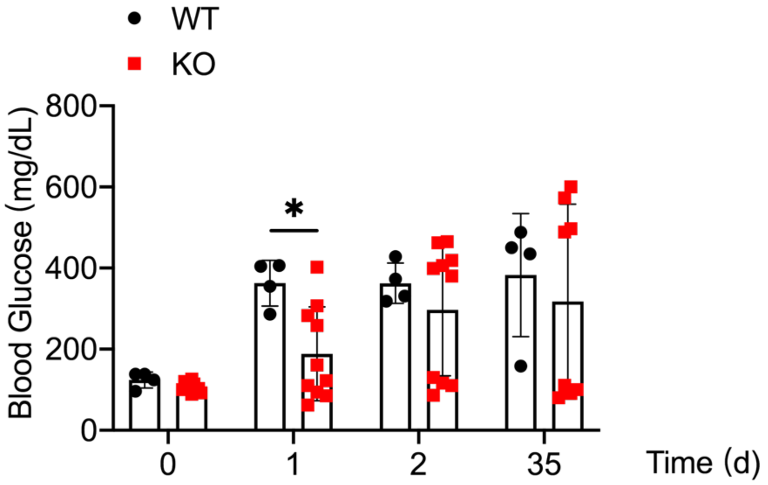

Supplemental Figure 2. Blood glucose levels of the high-dose STZ diabetic mouse model. WT: wildtype (n=4); KO: Mpv17 mutant (n=10). The comparison between the two groups was performed using two-tailed, unpaired Student’s t-test. *p<0.05, statistically significant.

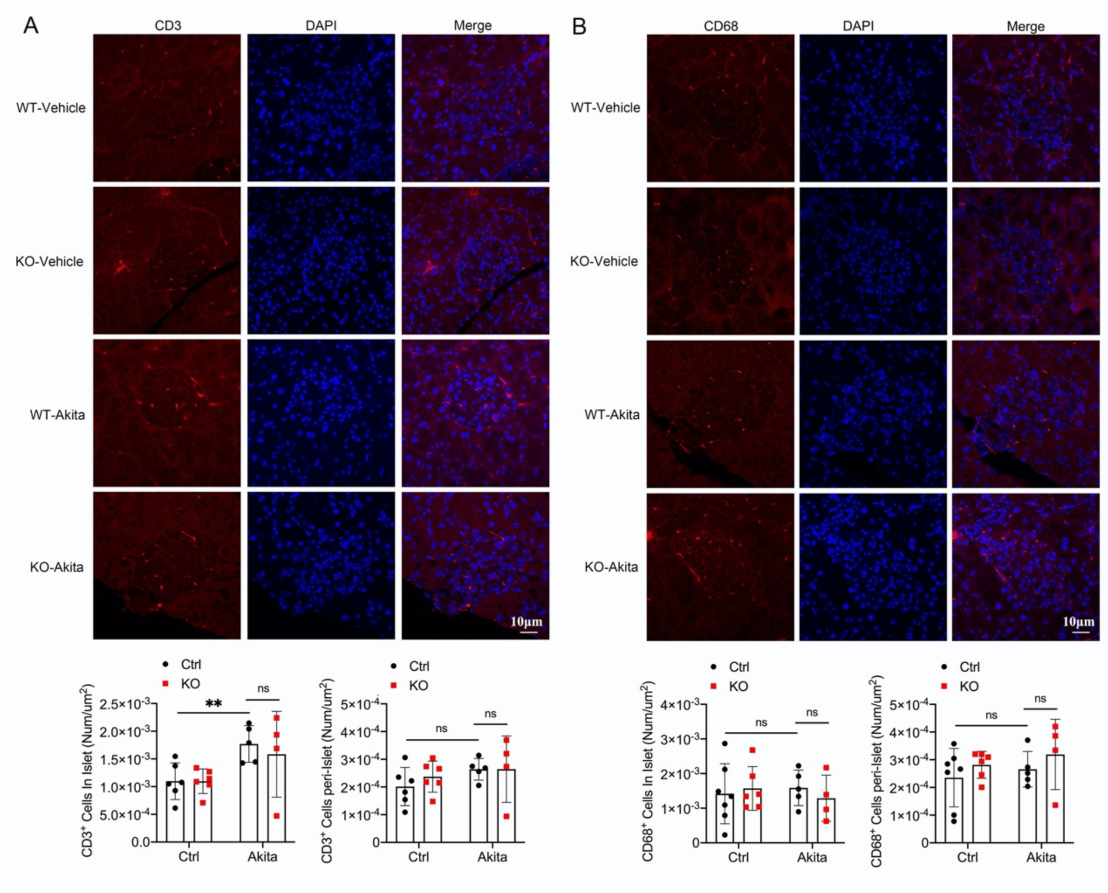

Supplemental Figure 3. CD3 and CD68 staining for analysis of $CD3^+$ (A) and $CD68^+$ cells (B) infiltration in islets and peri-islets in the mice described in Figure 2B. The results are presented with mean±SD of each group of mice. Comparisons between groups were performed using two-way ANOVA followed by Bonferroni post-hoc test. *$p<0.05$, statistically significant; **$p<0.01$, statistically very significant; ns: no significant difference.

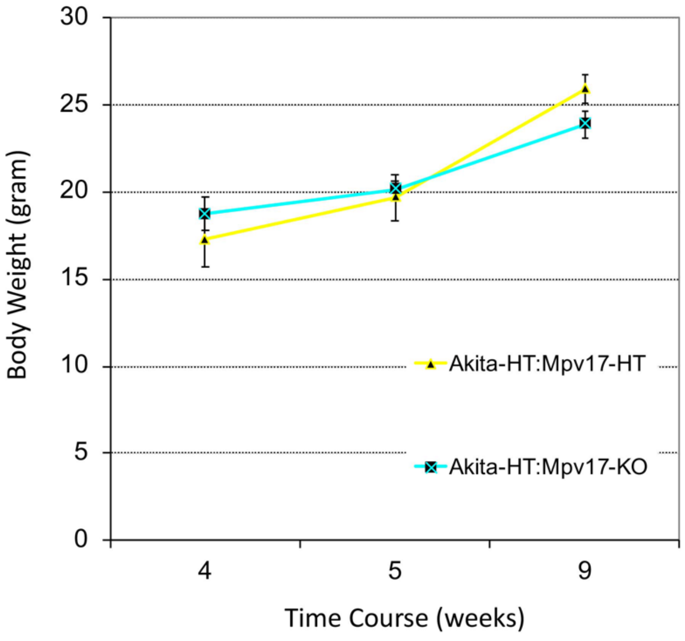

Supplemental Figure 4. Body weight of MPV17 mutant and control mice with Ins2^{Alita}. The comparison between the two groups was performed using two-tailed, unpaired Student's t-test.

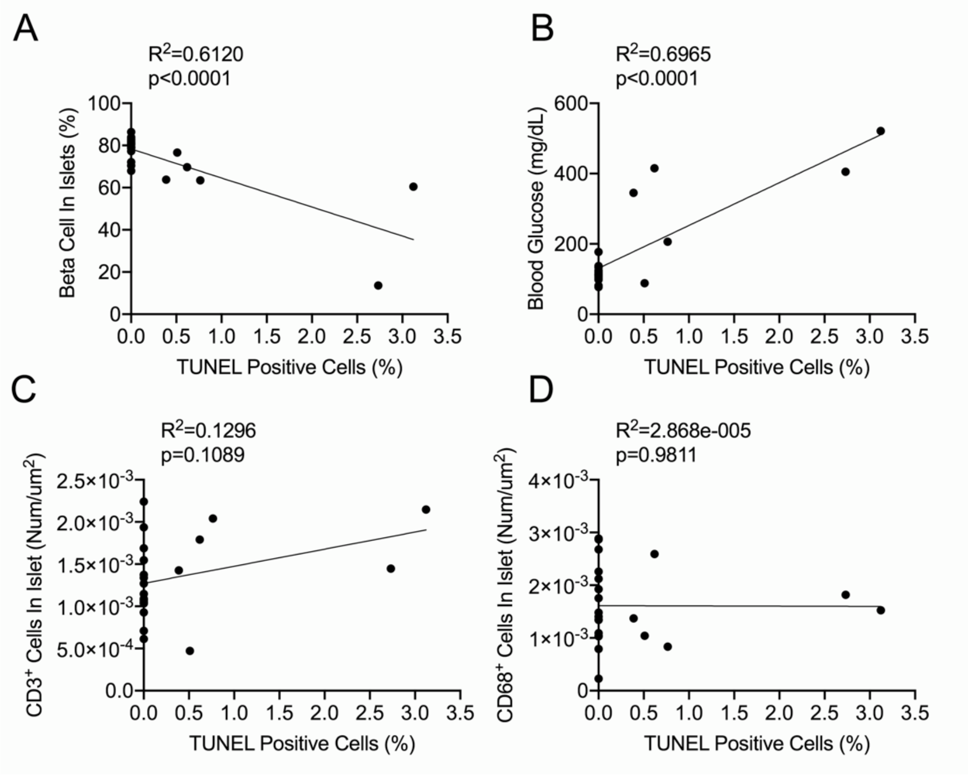

Supplemental Figure 5. Correlations between beta-cell apoptosis vs. beta-cell number (A), blood glucose (B), CD3+ cell number (C), and CD68+ cell number (D) in the STZ model.

Correlation analysis was performed using two-tailed correlations analysis, and R^2 was computed with each paired X with Y.

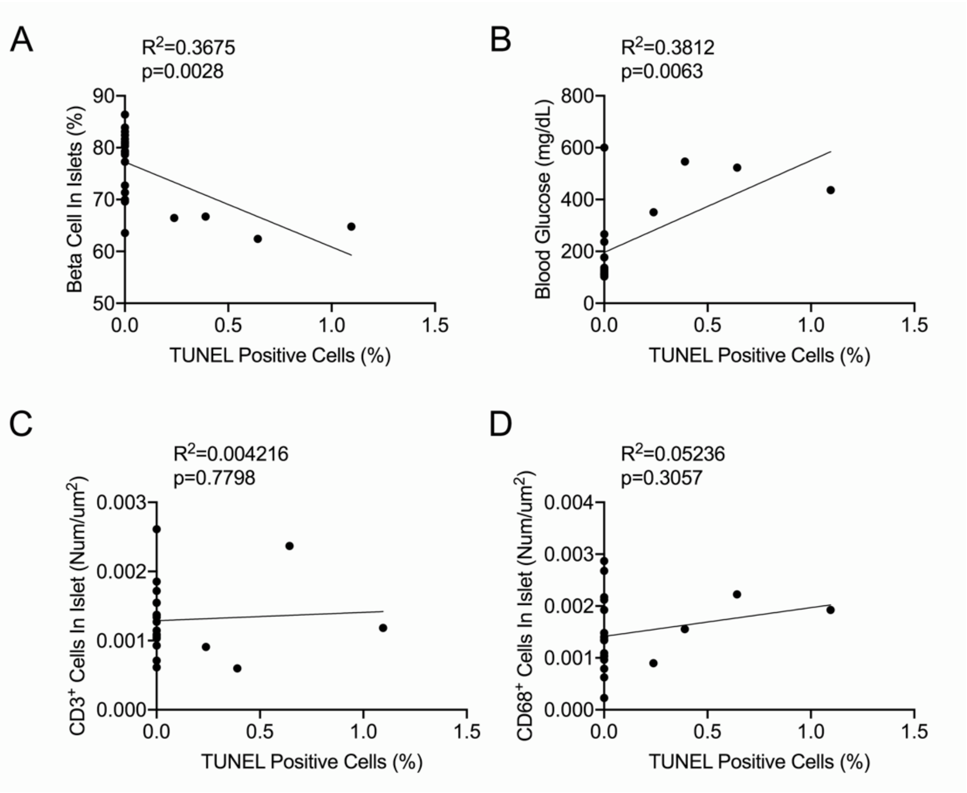

Supplemental Figure 6. Correlations between beta-cell apoptosis vs. beta-cell number (A), blood glucose (B), CD3+ cell number (C), and CD68+ cell number (D) in the Akita model. Correlations analysis was performed using two-tailed correlations analysis, and R^2 was computed with each paired X with Y.

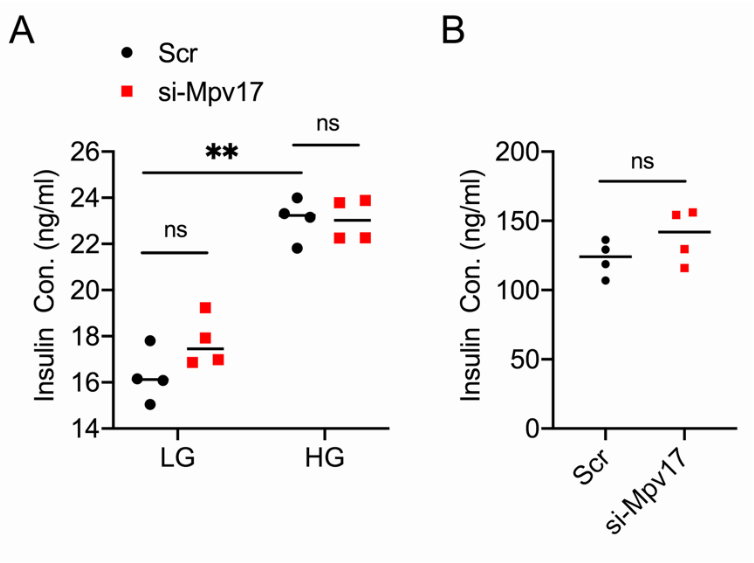

Supplemental Figure 7. The effect of MPV17 deficiency on insulin secretion of beta-cells. MPV17 deficiency did not affect insulin content in the supernatant of medium (A) and in the cell lysates (B). LG: low glucose (2.8 mM); HG: high glucose (16.7 mM). The result is presented with mean ± SD. Comparisons between groups were performed using two-way ANOVA followed by Bonferroni post-hoc test. **p<0.01, statistically very significant; ns: no significant difference.
